# Military Amputations. II. The Treatment of Stumps in Preparation for Artificial Limbs

**Published:** 1916-06-03

**Authors:** 


### MILITARY AMPUTATIONS.

### II. The Treatment of Stumps in Preparation for Artificial Limbs.

In the former article* the reasons were given for the very marked differences between the methods of amputation usual in civil practice since the establishment of the Listerian era, and those commonly pursued by military surgeons in the present war. It was pointed out that the saving of life by preventing or limiting suppuration and consequent blood-poisoning has necessitated a return to primitive methods of amputation. These methods have been forced upon the surgeons, much against their will, by the heavily infected nature of the wounds which they are called upon to treat; and they result in stumps which are on the average very much less suitable for the fitting of artificial limbs than those which result from the select types of amputation which civilian surgery had gradually evolved and improved. The upshot of this state of affairs is that many soldiers, when their amputation wounds are at length healed, are found to possess stumps which for one cause or another are unsuitable for the fitting of artificial limbs. Sometimes these disabilities can be remedied comparatively easily, sometimes only by a further operation, and sometimes they cannot be completely remedied at all.

### A Useful Memorandum.

Now, although the early Victorian circular or guillotine amputations are very often forced upon the surgeons by their experience of unsatisfactory results from less unæsthetic procedures, it by no means follows that a poor stump need necessarily accrue. Much can be done by intelligent care and forethought in after-treatment to minimise the inconveniences, from the surgical-instrument maker's standpoint, of a large wound healing by granulation. These inconveniences do not, of course, reveal themselves to the surgeons operating abroad, nor indeed do many of those in home hospitals fully appreciate them. Accordingly, a brief and very valuable memorandum has been prepared by War Office authority for distribution among military hospital staffs. Some of the hints on amputation work contained in it are as old as surgery itself: others are the outcome of the unique experience gained during the present war; but all are relevant and well worthy of the attention of those who are doing military surgery.

Thus the pamphlet opens with the familiar exhortation to avoid amputation through a joint, if possible; it is well to have so ancient and stereotyped a piece of advice reiterated, because the reason for it is mainly dependent on certain difficulties which such an amputation imposes upon the surgical-instrument maker; these difficulties have not been fully surmounted by any of the great advances which have taken place in this art; and, therefore, it is right that the fact should be made known that the old advice is still sound and valid to-day. Next follows a commendation of the circular or guillotine operation, *provided that it is followed by the neatest possible secondary amputation as soon as the wound is clean.* This is a most important stipulation, and one which is in many cases neglected. By adhering to it the surgeon can minimise almost completely the drawbacks to the subsequent utility of the limb which otherwise so frequently arise. Skin grafting is *not* a satisfactory substitute for this after-treatment by secondary amputation.

### The Selection of Partial Amputations.

The various sites of amputation are then taken in detail, with instructive comments on each. Thus it is pointed out that at the wrist-joint the usual objections to amputation through a joint do not hold good. The various partial amputations of the foot (Chopart's, Pirogoff's, Lisfranc's, and modifications of them) are condemned as all inferior to Syme's operation, which is preferred whenever anything more than mere removal of toes is necessary. In dealing with amputations very high up, close to or at the hip and shoulder, the definite statement is made that there is no case of amputation in either extremity in which an artificial limb cannot be fitted; but the great importance of leaving even two or three inches of the femur or humerus whenever possible is duly emphasised. Stress is also laid upon the fact that the arguments in favour of the "sites of election" for amputations have been partially invalidated by recent advances in the art of artificial-limb making. Nor is it so necessary as it used to be to avoid having the operation scar on the extremity of the stump. The reason for both these remarks is that the modern artificial limb often takes its bearing surfaces from well above the amputation site, instead of from the end of the stump, as was formerly almost universal.

The conditions which prevent or delay the fitting of an artificial limb are carefully considered; four months from the date of amputation is laid down as the normal period at which the fitting of a limb should be feasible. Sinuses, it is justly remarked, are nearly always due to a piece of dead bone; such sequestra can generally be detected by $x$-rays, and must be removed before healing will occur. Painful stumps are not always due to bulbous nerve ends: the latter should be prevented by care in operating, and if they occur must be removed. Other causes of painful stumps are indicated, together with the appropriate remedies. The importance and undesirability of contractures are pointed out, and stress is laid upon the responsibility of the surgeon in preventing these fertile sources of pain and of incapacity after amputations. Altogether the brief memorandum is an excellent achievement; if it is the outcome of a recent appointment as orthopædic expert to the Army, the holder has certainly already amply justified his selection for it.

* The previous article appeared on May 27.

